# Nutrient sensing in the nucleus of the solitary tract mediates non-aversive suppression of feeding via inhibition of AgRP neurons

**DOI:** 10.1016/j.molmet.2020.101070

**Published:** 2020-09-06

**Authors:** Anthony H. Tsang, Danae Nuzzaci, Tamana Darwish, Havish Samudrala, Clémence Blouet

**Affiliations:** Metabolic Research Laboratories, Wellcome Trust MRC Institute of Metabolic Science, Addenbrooke's Hospital, Hills Road, Cambridge, CB2 0QQ, UK

**Keywords:** Nucleus of the solitary tract, Appetite, Obesity, AgRP neurons, Hypothalamus, Metabolic diseases

## Abstract

The nucleus of the solitary tract (NTS) is emerging as a major site of action for the appetite-suppressive effects of leading pharmacotherapies currently investigated to treat obesity. However, our understanding of how NTS neurons regulate appetite remains incomplete.

**Objectives:**

In this study, we used NTS nutrient sensing as an entry point to characterize stimulus-defined neuronal ensembles engaged by the NTS to produce physiological satiety.

**Methods:**

We combined histological analysis, neuroanatomical assessment using inducible viral tracing tools, and functional tests to characterize hindbrain-forebrain circuits engaged by NTS leucine sensing to suppress hunger.

**Results:**

We found that NTS detection of leucine engages NTS prolactin-releasing peptide (PrRP) neurons to inhibit AgRP neurons via a population of leptin receptor-expressing neurons in the dorsomedial hypothalamus. This circuit is necessary for the anorectic response to NTS leucine, the appetite-suppressive effect of high-protein diets, and the long-term control of energy balance.

**Conclusions:**

These results extend the integrative capability of AgRP neurons to include brainstem nutrient sensing inputs.

## Highlights

• Leucine sensing in the NTS rapidly inhibits AgRP neurons.
• AgRP neurons receive polysynaptic inputs from multiple discrete hindbrain nuclei.
• NTS PrRP neurons sense leucine and project to AgRP neurons.
• NTS leucine sensing activates DMH LepR^+^ neurons that project to AgRP neurons.
• This circuit is required for the anorectic actions of NTS leucine and dietary proteins, and the regulation of energy balance

## Abbreviations

AP: area postrema
ARH: arcuate nucleus of the hypothalamus
DMH: dorsomedial nucleus of the hypothalamus
NTS: nucleus of the solitary tract
PBN: parabrachial nucleus
PrRP: prolactin-releasing peptide
PRV-Bartha: pseudorabies virus Bartha strain
PVH: paraventricular nucleus of the hypothalamus
RVLM: rostral ventrolateral medulla
TT: tetanus toxin
VMH: ventromedial nucleus of the hypothalamus
vsPVC: ventrocaudal part of the spinal trigeminal nucleus
4-OHT: 4-hydroxytamoxifen

## 1 Introduction

The nucleus of the solitary tract (NTS) is established as a major brain site for the sensing and integration of signals relevant to the control of feeding behavior. It is a neuroanatomical hub for ascending vagal afferents activated by ingested foods, corticolimbic-descending inputs encoding homeostatic, cognitive, and motivational controls of feeding, and blood-borne signals diffusing from the adjacent area postrema (AP) that lacks a blood-brain barrier [1,2]. Molecularly, it is enriched in specialized interoceptive neuronal populations equipped to monitor circulating levels of nutrients, gut hormones, and adiposity signals [3].

NTS processing of these diverse inputs is classically described as the main mediator of the short-term negative feedback control of ingestion (or satiation) via recruitment of medullary motor output circuits [1]. The NTS also relays processed information to the lateral parabrachial nucleus (lPBN), established as a common target for NTS efferents in the central representation of aversive and avoidance feeding-related cues [4,5]. In both cases, the NTS outputs interrupt food ingestion and until recently had not been implicated in the regulation of hunger, the long-term control of satiety, or hedonic feeding. Studies applying molecular genetics or modern circuit analysis tools to the functional characterization of NTS neurons revealed that the NTS can in fact modulate a much larger range of behavioral effectors of energy balance including meal initiation and satiety [3,[6], [7], [8], [9]]. However, little is known about the neural mechanisms through which the NTS regulates forebrain hunger and satiety circuits and the physiological contexts in which these NTS feeding-regulatory forebrain-projecting outputs are engaged.

Conceptually, a key question is whether different behavioral effectors of ingestion (that is, satiation, avoidance/aversion, satiety, and food-seeking) are engaged by distinct and functionally specialized NTS neuronal subsets. Evidence that segregated subsets of CCK^NTS^ or TH^NTS^ neurons project to the lPBN or hypothalamus to produce either avoidance/aversive anorexia vs satiety or glucoprivic feeding support this view [10,11]. Alternatively, or in addition to this possibility, recruitment of the same neurons could simultaneously or gradually induce many of these behavioral outputs. The fact that NTS catecholaminergic neurons send collaterals to midbrain and forebrain targets provides a neuroanatomical basis for the latter [12], which could explain the ability of high doses of satiation hormones such as CCK to recruit aversive circuits [13] and/or provide a mechanism for the synergistic feeding suppressive effects produced by the combination of anorectic signals [3,14,15]. Addressing this question with molecularly defined circuit analysis tools is difficult because most identified NTS neurochemical subsets are functionally heterogeneous, respond to multiple cues, and project widely throughout the neuraxis [7,16]. Instead, it may be possible to better understand the functional organization of NTS feeding-regulatory circuits using functionally defined circuit mapping, which could be particularly insightful if subsets of NTS neurons are specialized in the transmission of highly specific sensory cues and organized in a similar fashion as gustatory and vagal sensory neurons [17,18]. Applying such a strategy to signals able to produce satiation or satiety without negative consequences may lead to an important new understanding of how to pharmacologically suppress appetite without undesirable side effects.

We previously showed that NTS sensing of the branched-chain amino acid leucine not only modulates the control of meal size, but also rapidly suppresses hunger in fasted animals and increases satiety without the production of conditioned taste aversion [19,20]. In this study, we used NTS leucine sensing as a functional entry point to investigate ascending neural circuits engaged by NTS neurons to modulate hunger and satiety. In these experiments, leucine is injected into the NTS at physiologically relevant doses to model the postprandial increase in brain leucine levels seen in response to the consumption of high-protein meals, a dietary paradigm that potently suppresses food intake. We employed an activity-dependent labeling and circuit mapping strategy that allowed the expression of circuit analysis tools specifically in leucine-sensing neurons and downstream circuits.

## 2 Materials and methods

### 2.1 Experimental models

All the experiments were conducted on male mice in accordance with the Animals (Scientific Procedures) Act 1986 and approved by the local animal ethics committees. The mice were obtained from Charles River UK (8-week-old C57/bl6J) or the Jackson Laboratories (*Agrp-ires-cre*, *Th-cre*, and *NPY-hrGFP*), housed in individually ventilated cages with standard bedding and enrichment, and maintained in a humidity-controlled room at 22–24 °C on a 12 h light/dark cycle with ad libitum access to water and standard laboratory chow diet unless otherwise stated. Isocaloric modified diets with varying protein amounts were custom made by Research Diets as per the formulations in Suppl. Table 1. For all the experiments using Cre reporter lines, we conducted the work in hemizygous males or wild-type littermates randomly assigned to the experimental groups. For studies on wild-type mice, weight-matched groups were compared. Before each dietary change, the mice were briefly exposed to the new diets to avoid neophobia or other novelty-related responses in subsequent experiments.

### 2.2 Stereotaxic surgical procedures

Surgical procedures were conducted on 9- to 11-week-old mice under isoflurane anesthesia. All the animals received Metacam prior to surgery and 24 h after surgery and were allowed a 1-week recovery period during which they were acclimatized to injection procedures. The mice were stereotactically implanted with bilateral steel guide cannulas (Plastics One) positioned 1 mm above the ARH (A/P: 1.1 mm, D/V: 4.9 mm, and lateral: +/−0.4 mm from the bregma) or the DMH (A/P: 1.5 mm, D/V: 4 mm, lateral: and +/−0.4 mm from the bregma) or 2 mm above the caudomedial NTS (cannula-holding bar in a 10° rostro-caudal angle with coordinates relative to the occipital suture: A/P: +0.5 mm, D/V: 3 mm, and lateral: +/−0.4 to the midline). Beveled stainless steel injectors (33 gauge) extending 1 mm (for ARH and DMH) and 2 mm (for NTS) from the tip of the guide were used for injections. For chronic cannula implantation, the cannula guide was secured in place with Loctite glue and dental cement (Fujicem2). Correct targeting was confirmed histologically postmortem. The mice were allowed 1 week of recovery during which they were handled daily and acclimatized to the relevant experimental settings.

### 2.3 Viral vectors and injection procedures

For Cre-dependent retrograde polysynaptic tracing, we used PRV-Introvert, a newly developed version of PRV-Bartha in which retrograde viral propagation and reporter expression are activated only after exposure to Cre recombinase with high specificity [21], kindly provided by Prof. Jeff Friedman (Rockefeller University). PRV-Introvert was prepared as previously described [21]. Virus stocks were grown and tittered in PK15 cells (7.89 × 10^8^ pfu/ml) (ATCC). Viral specificity was tested in vitro in HEK cells transfected with Cre and by stereotaxic injection into the ARH of wild-type mice (n = 5). A total of 25 mice were used to characterize polysynaptic inputs to AgRP neurons. All received 100 nl of PRV-Introvert into the ARH and were sacrificed at 0, 24, 48, 72, or 96 h after injection. The mice rapidly developed symptoms, were closely monitored, provided with hydrogel, mash, and a heating pad throughout the postsurgical period, and killed before reaching 20% of presurgical weight loss.

Retrograde Cre-dependent monosynaptic tracing was performed using AAV8-hSyn-FLEX-TVA-P2A-eGFP-2A-oG (2.82 × 10^12^ vg/ml, 500 nl per side) and the modified rabies strain SADΔG-mCherry(EnvA) (1.1 × 10^9^ TU/ml, 100 nl bilaterally into the ARH 3 weeks later) [22,23] both obtained from the Salk Institute Viral Vector Core. A total of 23 mice were used and perfused at different survival times after the rabies injection and up to 2 weeks later.

Activity-dependent cell labeling and anterograde tracing were performed using the viral construct AAV8-fos-Cre-ERT2-PEST (AAV-fos-CreER, 8.8 × 10^12^ vg/ml, 300 nl per side bilaterally, donated by Prof. Deisseroth via the Stanford Virus Core) combined with AAV8-EF1a-DIO-hChR2(H134R)-mCherry (AAV-DIO-ChR2: mCherry, 1.9 × 10^1^³ vg/ml, 300 nl per side, Addgene) or AAV-DJ-CMV-DIO-eGFP-2A-TeNT (AAV-DIO-TeNT, 5.13 × 10^12^ vg/ml, 300 nl per side, Stanford University Neuroscience Gene Vector and Virus Core).

### 2.4 NTS leucine injection and acute food intake assessments

Studies were conducted in a home cage environment. For NTS leucine injection, the mice were food deprived for 6 h during the day before receiving a bilateral parenchymal injection of l-leucine (Sigma, 2.1 mM, 50 nl/side, and 50 nl/min) or aCSF (R and D) and either immediately returned to their home cage for food intake analysis or perfused 80/90 min later for histological assessments. For food intake studies, the injection occurred 1 h before dark onset. The mice were refed after the injection and their food intake was monitored over various time points after refeeding. For the meal initiation experiment, digital cameras were used to record the first 30 min of feeding response after the mice received the brain injection and were provided a food pellet. All the studies were conducted in a cross-over randomized manner on age- and weight-matched groups, and at least 4 days elapsed between each brain injection.

### 2.5 Activity-dependent induction of cre expression

The mice that received AAV-fos-CreER into the NTS or DMH and were chronically equipped with a cannula guide targeting the NTS and underwent a series of induction sessions as follows. The mice received an injection of leucine into the NTS in their home cage as previously described and 80 min later were dosed with tamoxifen metabolite 4-hydroxytamoxifen (4-PHT, Sigma, 40 mg/kg i.p.) prepared using a formulation previously described [24]. The mice remained fasted for 4 h after the 4-HT injection. Each induction session was separated by a minimum of 96 h.

### 2.6 Brain perfusion, immunohistochemistry, microscopy, and image analysis

The animals were anaesthetized with Dolethal (Vetoquinol UK Ltd.) at 1 ml/kg in saline and transcardially perfused with 0.1 M heparinized PBS followed by 4% paraformaldehyde. Brains were extracted and post-fixed in 4% paraformaldehyde and 30% sucrose for 48 h at 4 °C. The brains were sectioned using a Leica freezing sliding microtome into 5 subsets of 25 micron sections. Antigen retrieval was used for all the experiments prior to antibody incubation. Sections were incubated in 10 mM sodium citrate at 80 °C for 20 min and then washed three times in PBS. Tissue was blocked for 1 h with 5% normal donkey serum or 5% normal goat serum (Abcam) at room temperature and incubated at 4 °C with primary antibodies against c-Fos (1:2000, Synaptic Systems, Cat# 226003; RRID:AB_2231974) [25], dsRed (1:1000, Clontech, Cat# 632392; RRID:AB_2801258) [26], GFP (1:1000, Abcam, Cat# ab13970; RRID:AB_300798) [27], TH (1:200, Cat# 22941; RRID:AB_572268, Immunostar) [28], PrRP (1:1000, Abcam, Cat# ab251707), Ha (1:1000, Cell Signaling Technology, Ca# 3724; RRID:AB_1549585) [29] GFRAL (1:200, Thermo Fisher Scientific, Cat# PA5-47769; RRID:AB_2607220), and SYP (1:500, Synaptic Systems, Cat# 101 011; RRID:AB_887824) [30]. The sections were then mounted on slides and coverslipped with Prolong Diamond (Thermo Fisher Scientific).

The sections were imaged using a Zeiss Axio slide scanner with a 20x objective or a Leica SP8 confocal microscope with 40x or 63x objectives. The imaging settings remained the same between the experimental and control conditions.

Images of tissue sections were digitized, and areas of interest were outlined based on cellular morphology and using Paxinos and Franklin's brain atlas [31]. The images were analyzed using the ImageJ manual cell counter or Zeiss ZEN 2.3 software. To assess projection coverage to the ARH, Imaris software (Oxford Instruments plc) was used to 3D reconstruct the ARH image stacks acquired by the SP8 microscope and analyze the contact areas.

### 2.7 Multiplexed FISH with RNAscope

The mice were perfused as previously described. Their brains were post-fixed in 4% PFA solution overnight and then cryoprotected in 30% sucrose solution in PBS for up to 24 h. The tissue was covered with optimal cutting temperature (OCT) media and then sliced to 16 μm thick using a Leica CM1950 cryostat directly onto Superfrost Plus slides (Thermo Fisher Scientific) in an RNase-free environment. The slides were then stored at −80 °C.

Multiplexed fluorescence in situ RNA hybridization (FISH) was performed using RNAscope technology. After epitope retrieval and dehydration, the sections on the slides were processed for multiplexed FISH using an RNAscope LS Multiplex Assay (Advanced Cell Diagnostics). The samples were first permeabilized with heat in Bond Epitope Retrieval solution 2 (pH 9.0, Leica AR9640) at 95 °C for 2 min, incubated in protease reagent (Advanced Cell Diagnostics) at 42 °C for 10 min, and then treated with hydrogen peroxide for 10 min to inactivate endogenous peroxidases and the protease reagent. The samples were then incubated in z probe mixtures for 2 h at 42 °C and washed 3 times. DNA amplification trees were built through incubations in AMP1 (preamplifier), AMP2 (background reducer), and then AMP3 (amplifier) reagents (Leica) for 15–30 min each at 42 °C. Between incubations, the slides were washed with LS Rinse buffer (Leica). The samples were then incubated in channel-specific horseradish peroxidase (HRP) reagents for 15 min at 42 °C, TSA fluorophores for 30 min, and HRP blocking reagent for 15 min at 42 °C. The following TSA labels were used to visualize the z probes: Cy3 (1:500), FITC (1:500), and Cy5 (1:500) fluorophores (PerkinElmer).

The brain sections were imaged using a spinning disk Operetta CLS (PerkinElmer) in confocal mode using an sCMOS camera and a 40x automated water-dispensing objective. The sections were imaged with z stacks at intervals of 1 μm. ROIs included the PVH, DMH, NTS, AP, and DMX. Gain and laser power settings remained the same between the experimental and control conditions during each experiment. Harmony software (PerkinElmer) was used to automatically quantify the number of labeled RNA molecules (spots) per cell and the number of labeled cells among other metrics.

### 2.8 Statistical analysis

All the data presented as means ± SEM were analyzed using GraphPad Prism 8. For all of the statistical tests, an α risk of 5% was used to define statistical significance. Dietary and aCSF/leucine treatments where allocated randomly in weight-matched groups. When possible, we performed within-mice comparisons and treatments were delivered in a cross-over manner in weight-matched groups. All the kinetics were analyzed using repeated-measures two-way ANOVAs and adjusted with Bonferroni's post hoc tests. Multiple comparisons were tested with one-way ANOVAs and adjusted with Tukey's post hoc tests. Single comparisons were conducted using two-tailed student's t tests. We used blinding (to mouse genotype, viral treatment, or drug delivered) for in vivo experiments and to conduct image analyses. Additional statistical details on each experiment can be found in the figures or figure legends.

## 3 Results

### 3.1 NTS amino acid sensing inhibits AgRP neurons via a polysynaptic circuit

**Figure 1:**
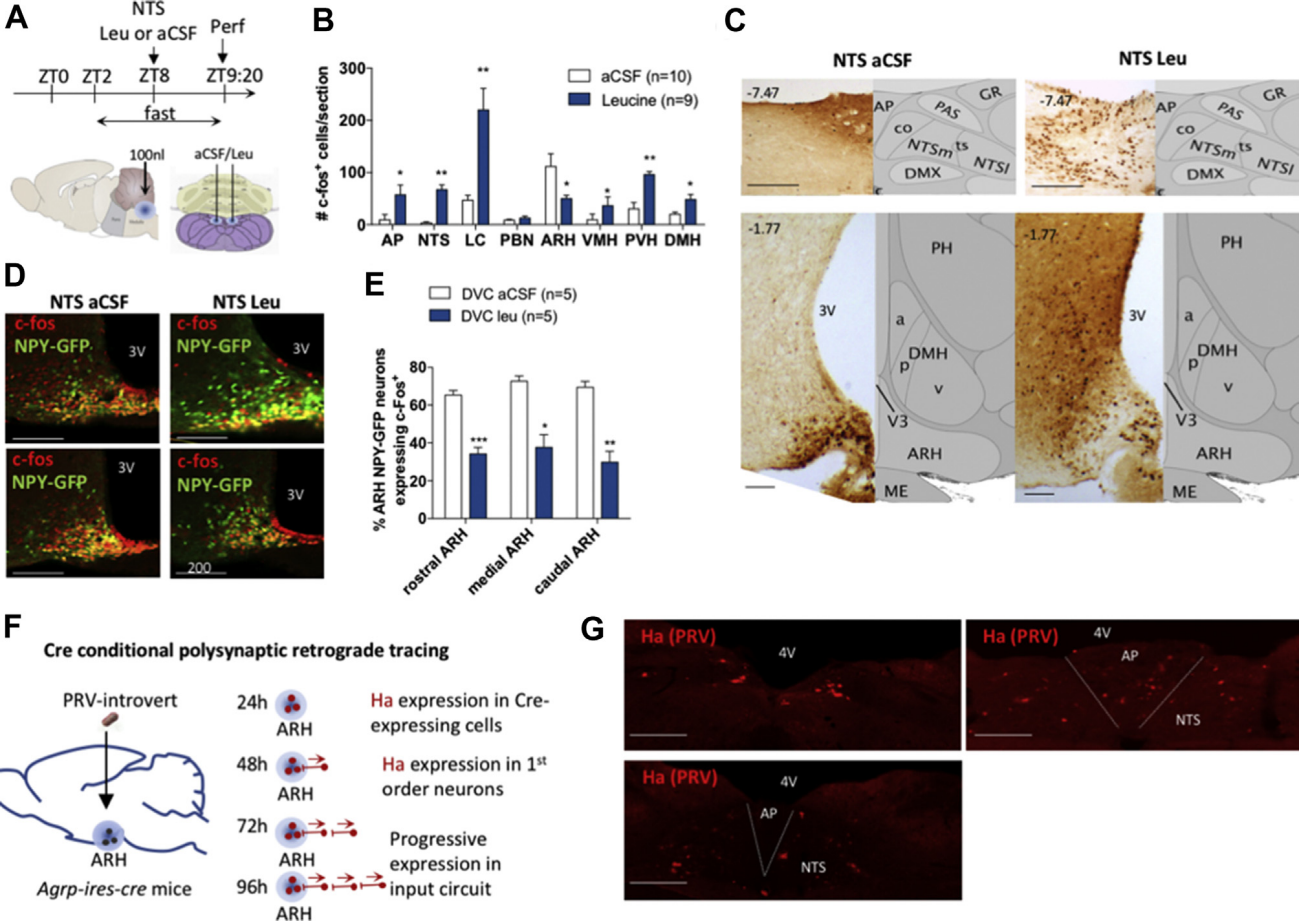
NTS amino acid sensing inhibits AGRP neurons via a polysynaptic circuit. Injection paradigm (a) used to activate NTS leucine-sensing neural circuits in mice. Quantification of c-Fos immunolabeling (b) and representative images (c) from selected sites of the mouse brain following NTS leucine delivery. Representative images (d) and quantification (e) of c-Fos immunodetection in AgRP/NPY neurons in the ARH. Protocol for Cre conditional polysynaptic retrograde tracing using PRV-Introvert (f) and expression of the PRV-Introvert reporter Ha in the caudomedial NTS after 96 h (g). Scale bar = 200 μm. AP: area postrema, NTS: nucleus of the solitary tract, LC: locus coeruleus, PBN: parabrachial nucleus, ARH: arcuate nucleus of the hypothalamus, VMH: ventromedial nucleus of the hypothalamus, PVH: paraventricular nucleus of the hypothalamus, DMH: dorsomedial nucleus of the hypothalamus, 3V: third ventricle, 4V: fourth ventricle. Rostral ARH: bregma −1.07 to −1.37, medial ARH: bregma −1.37 to −1.77, caudal ARH: bregma −1/77 to-2.07. ∗p < 0.05 vs aCSF, ∗∗p < 0.01 vs aCSF, and ∗∗∗p < 0.001 vs aCSF. All the results are shown as means ± SEM.

NTS leucine sensing rapidly reduces hunger in fasted rodents [3,32], but the underlying neural circuit mediating this response in unknown. To characterize the ascending neural circuits engaged by NTS leucine sensing to rapidly inhibit appetite, we first assessed neuronal activation throughout the neuraxis in response to local bilateral NTS leucine administration. Mice were fasted for 6 h and received a site-specific injection of 50 nl of leucine per side into the caudomedial NTS as previously described [32] (Figure 1A). Neuronal activity was assessed using c-Fos immunohistochemistry 80 min later. NTS leucine induced robust c-Fos expression in the caudomedial NTS as well as in the adjacent area postrema (AP) (Figure 1B,C). Outside this region, only a few brain sites were significantly activated by local NTS leucine administration compared to aCSF vehicle: the locus coeruleus (LC) and the paraventricular, ventromedial, and dorsomedial nuclei of the hypothalamus (Figure 1B,C). In contrast, NTS leucine produced a ∼50% decrease in c-Fos immunolabeling in the ARH (Figure 1B,C). Of note, NTS leucine did not produce neuronal activation in the parabrachial nucleus (PBN, Figure. 1B), consistent with the lack of conditioned avoidance in response to parenchymal NTS leucine administration in the mice [20].

The ARH contains intermingled orexigenic and anorexigenic neurons including AgRP neurons, which are critical for the development of food-seeking behavior and meal initiation in hungry mice [33,34]. We previously found that NTS leucine sensing robustly increases first-meal latency in fasted mice, hence reducing the drive to approach and consume food [3,32]. This, together with the reduced c-Fos expression in the ARH following NTS leucine administration, prompted us to hypothesize that hindbrain leucine sensing may rapidly inhibit AgRP neurons. To test this, we repeated the same experiment (Figure. 1A) in the Npy*-hrGFP* transgenic mice, where the hrGFP signal in the mediobasal hypothalamus selectively labels all AgRP neurons [35]. A majority of ARH NPY/AgRP neurons were activated under control conditions (Figure 1D,E). As predicted, NTS leucine produced a ∼50% decrease in NPY/AgRP neuronal activation throughout the rostro-caudal extent of the ARH (Figure 1D,E). Thus, NTS leucine sensing rapidly inhibits ARH NPY/AgRP neurons.

Previous research indicated that NTS inputs can modulate the activity of AgRP neurons [11,20], but the neuroanatomical organization of these inputs and the physiological conditions in which they are engaged to modulate feeding remain unclear. To establish that AgRP neurons are synaptically connected to NTS neurons, we conducted a series of retrograde viral tracing studies. Pseudorabies virus Bartha strain (PRV-Bartha) is a neuroanatomical tracer that is transmitted retrogradely across synapses and can be used to define polysynaptic inputs to infected neurons [36]. PRV-Introvert is a newly developed version of PRV-Bartha in which retrograde viral propagation and reporter expression are activated only after exposure to Cre recombinase with high specificity [21]. We used PRV-Introvert in *Agrp-ires-cre* mice to serially label chains of presynaptic neurons projecting to ARH AgRP neurons. The mice were sacrificed 0, 24, 48, 72, or 96 h after local ARH inoculation with PRV-Introvert and their brains were examined for HA reporter expression (Figure. 1F). As expected, we did not detect any HA immunolabeling in the brains of wild-type mice injected with the virus (Suppl. Fig. 1a), confirming Cre dependency. At 24 h after injection, PRV-Introvert was detectable in the ARH, indicating that Cre-mediated recombination occurred locally within 24 h of PRV injection. After 48 h, spread of the Cre-activated PRV virus was observed in multiple hypothalamic sites including the arcuate, ventromedial, dorsomedial, lateral, and paraventricular nuclei (Suppl. Fig. 1b and 1c). After 72 h, the medial amygdala was labeled (Suppl. Fig. 1d). After 96 h, we detected PRV in several pontine, midbrain, and hindbrain structures. These included the medial parabrachial nucleus (mPBN) (Suppl. Fig. 1e), the ventrocaudal part of the spinal trigeminal nucleus (vcSPVC) (Suppl. Fig. 1f), the rostral ventrolateral medulla (RVLM) (Suppl. Fig. 1e), the AP and NTS, both in its rostral portion and in the lateral portion of the caudomedial NTS (Figure 1G and Suppl. Fig. 1f–1g). Thus, ARH AgRP neurons receive inputs from multiple midbrain and hindbrain sites, including the caudomedial NTS.

The long survival time necessary to detect the presence of PRV in the NTS suggests that the NTS → AgRP circuit contains more than 1 synapse. Alternatively, the long distance that the virus needs to travel to label hindbrain sites may also explain the lack of signals at 48 and 72 h. To clarify this, we performed Cre-dependent monosynaptic retrograde viral tracing in the *Agrp-ires-cre* mice using an envelope protein (EnvA) pseudotyped glycoprotein (g)-deleted rabies virus modified to express mCherry (SADΔG-mCherry) (EnvA) [22,23]. AgRP Cre-expressing neurons were first genetically modified to co-express TVA (a receptor for the avian sarcoma leucosis virus glycoprotein EnvA) and oG (optimized rabies envelope glycoprotein) via targeted unilateral injections of rAAV8-hSyn-FLEX-TVA-P2A-eGFP-2A-oG into the ARH (Suppl. Fig. 1h). AgRP neurons infected with this construct became selectively competent for transduction by SADΔG-mCherry(EnvA) and expressed eGFP. Three weeks later, the mice received a unilateral injection of SADΔG-mCherry(EnvA) in the same injection site and were sacrificed at various survival times (up to 14 days). The brains were processed to examine mCherry expression. Two weeks after the SADΔG-mCherry(EnvA) injection, we observed dense eGFP expression in the ARH (Suppl. Fig. 1i) together with dense mCherry immunolabeling in the ARH, DMH, and PVH (Suppl. Fig. 1i and 1j). Overall, 42% of AgRP neurons expressing eGFP co-expressed mCherry. We carefully examined the NTS of 8 successfully infected animals throughout the rostro-caudal extent of the NTS but did not detect rabies-infected cell bodies (Suppl. Fig. 1k). These data support the conclusion that the NTS does not send monosynaptic inputs to AgRP neurons.

### 3.2 NTS PrRP neurons are leucine-sensing and project to AgRP neurons

**Figure 2:**
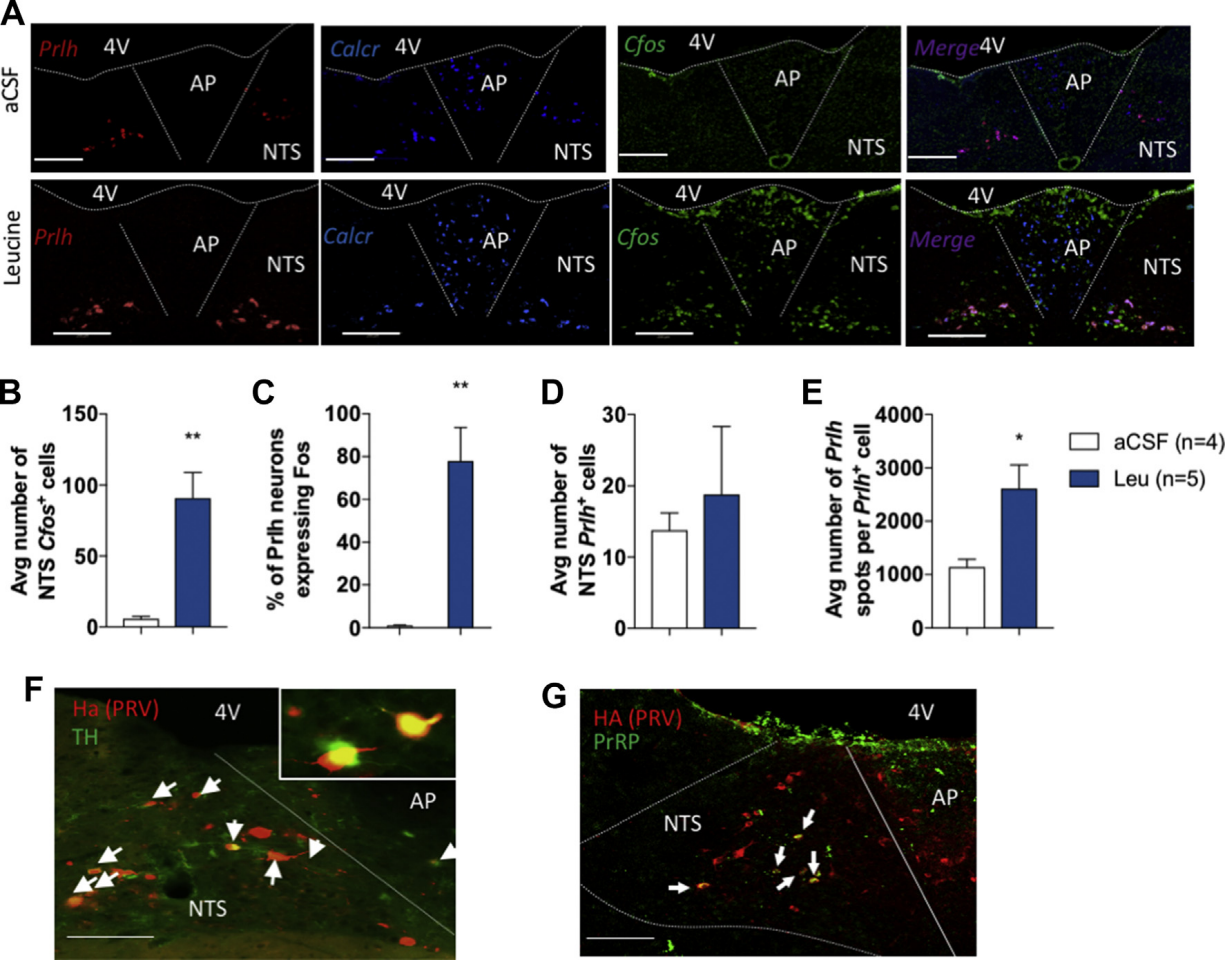
Neurochemical characterization of NTS leucine-sensing neurons. Representative images (a) and quantification (b–e) of multiplexed in situ hybridization against *Prlh*, *Calcr*, and *Cfos* in the dorsal vagal complex of mice after an injection of leucine into the NTS. Representative images of the colocalization of the Ha reporter from the polysynaptic retrograde virus PRV-Introvert and tyrosine hydroxylase (TH, f) or prolactin-releasing peptide (PrRP, g) by multiplexed immunofluorescent labeling in the NTS of the *Agrp-ires-cre* mice 96 h after PRV-Introvert delivery into the ARH. Scale bar: 200 μm. 4V: fourth ventricle, AP: area postrema, NTS: nucleus of the solitary tract. ∗p < 0.05 vs aCSF, ∗∗p < 0.01 vs aCSF, and ∗∗∗p < 0.001 vs aCSF. All the results are shown as means ± SEM.

Previous research showed that a majority of NTS leucine-sensing neurons express TH [3,32], but TH labels a molecularly and functionally diverse group of neurons, prompting us to further analyze the neurochemical identity of NTS neurons responsive to leucine. The NTS contains several neuronal subpopulations responsive to aversive gastrointestinal stimuli or nutritional stress, leading to the formation of visceral malaise, taste aversion, or avoidance [22,23,[37], [38], [39]]. In contrast, some NTS neuronal subtypes are recruited preferentially in response to physiological satiation cues and do not produce aversive anorexia even in the context of pharmacological activation. These include subsets of TH^NTS^ neurons expressing prolactin-releasing peptide (PrRP) [40,41] and recently characterized Calcr^NTS^ neurons [20]. We previously showed that NTS leucine does not produce conditioned avoidance [20], leading us to hypothesize that leucine specifically engages either PrRP^NTS^ or Calcr^NTS^ neurons to suppress feeding. To examine this possibility, we used RNAscope multiplex in situ hybridization (ISH) against *Fos*, *Prlh* (transcript for PrRP), and *Calcr* in caudomedial hindbrain sections of mice that received local NTS leucine injections as previously described (Figure. 1A). We found a significant overlap between PrRP^NTS^ and Calcr^NTS^ neurons (Figure 2A): 75.1 ± 1.3% PrRP^NTS^ neurons expressed *Calcr*, while 68.3 ± 0.9% Calcr^NTS^ neurons expressed *Prlh*. Most of the NTS *Calcr*^*+*^*/Prlh*^−^ neurons where concentrated in the dorsal NTS, and *Calcr* was also expressed in a dense *Prlh*^−^ neuronal population in the AP (Figure. 2A). Consistent with c-Fos immunolabeling, we found that *Fos* expression rapidly increased in the NTS and AP in response to local leucine delivery (Figure 2A–B). Leucine activated on average 80% of the PrRP^NTS^ neurons (Figure 2A,C), which represented 34.7 ± 8.6% of the total population activated by leucine in the caudomedial NTS. Analysis of high-resolution ISH images revealed that the number of PrRP^NTS^ neurons was similar between conditions (Figure. 2D), but leucine increased the expression of *Prlh* (Figure. 2E), introducing a role for PrRP neurotransmission in leucine-sensing neurocircuits.

We then determined whether PrRP^NTS^ neurons project to ARH^AgRP^ neurons using brain sections from the *Agrp-ires-cre* mice infected with PRV-Introvert and killed 96 h after infection. We found that a majority of the HA^+^ neurons labeled 96 h after ARH PRV-Introvert delivery colocalized with TH (61 ± 8%) and PrRP (35 ± 2%) (Figure 2F,G), confirming that PrRP^NTS^ neurons project to ARH^AgRP^ neurons. Of note, NTS Ha^+^ neurons did not express GDF15 receptor GFRAL (Suppl. Fig. 2a), indicating that GDF15 does not engage the NTS → AgRP^ARH^ circuit to suppress feeding.

### 3.3 NTS leucine sensing activates DMH LepR^+^/GPR10 neurons projecting to AgRP neurons

**Figure 3:**
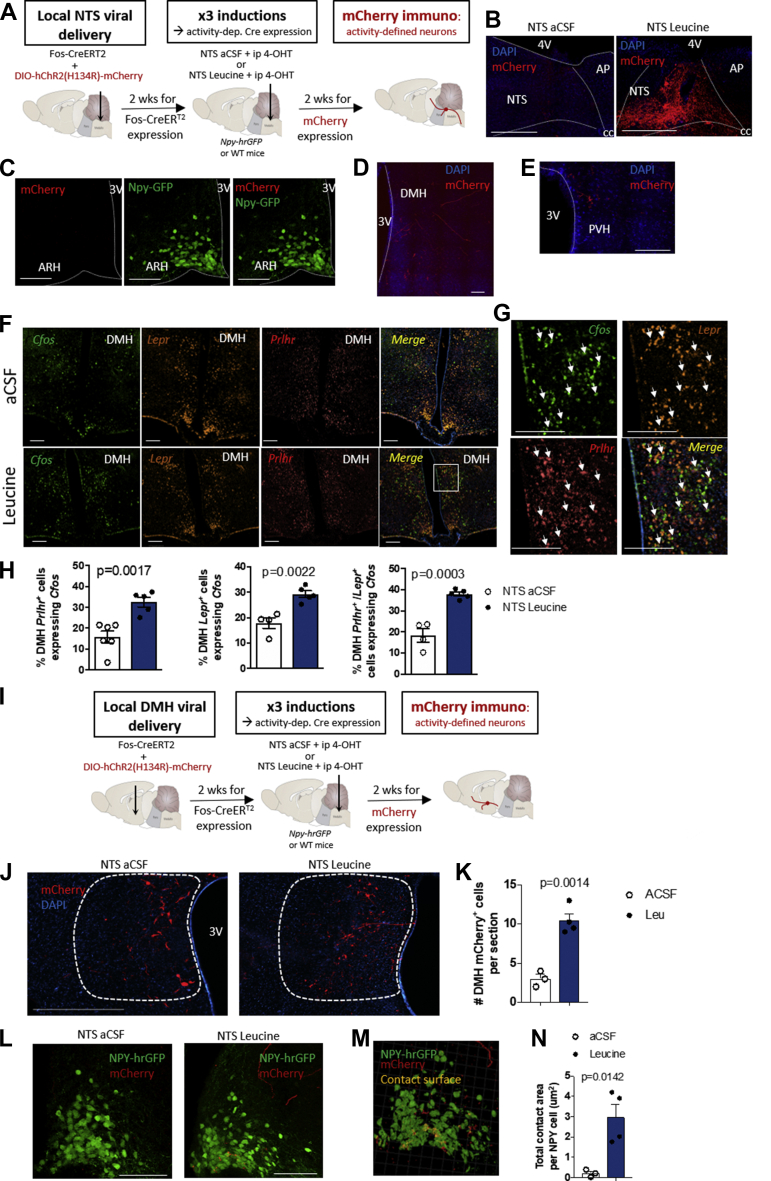
NTS leucine sensing activates DMH LepR^+^/GPR10 neurons projecting to AgRP neurons. Diagram of the experimental paradigm (a) and representative images of mCherry immunolabeling in the DVC (b), ARH (c), DMH (d), and PVH (e) for activity-dependent mapping of projection outputs of NTS leucine-sensing neurons using AAV-Fos-CreERT2 and AAV8-EF1a-DIO-hChR2(H134R)-mCherry viruses. Representative images (f), high-magnification images (g), and quantifications (h) of the expression of *Fos* in *Lepr*^*+*^ and *Prlhr*^*+*^ neurons in the DMH of the mice following an injection of aCSF or leucine in the NTS. Diagram of the experimental paradigm of activity-dependent labeling of DMH neurons responsive to NTS leucine sensing (i) and representative images (j and l), quantification (k and n), and Imaris 3D reconstruction (m) of mCherry immunolabeling in the DMH (j–k) and ARH (l–n) of the *Npy-hr-GFP* mice following injection of AAV-Fos-CreERT2 and AAV8-EF1a-DIO-hChR2(H134R)-mCherry viruses in the DMH and inductions with NTS aCSF or leucine. Scale bar is 200 μm. All of the results are shown as means ± SEM.

We next investigated the neuronal populations relaying NTS leucine-sensing inputs to AgRP neurons. Given that PrRP^NTS^ neurons represent only one-third of NTS leucine-sensing neurons and one-third of NTS neuron projection to AgRP^ARH^, and in the absence of a known specific molecular marker for NTS leucine-sensing neurons, we developed a strategy of activity-dependent circuit mapping following NTS leucine administration. We used the AAV8-Fos-ERT2-Cre-ERT2-PEST (AAV-Fos-CreERT2) virus to translate temporally delimited neuronal activity into sustained reporter expression [24]. Neurons expressing AAV-Fos-CreERT2 do not express Cre unless acutely exposed to an activating stimulus together with tamoxifen. A low dose of the tamoxifen metabolite 4-hydroxytamoxifen (4-OHT) allows genetic labeling of transiently activated neurons with high temporal specificity and low background [24]. The *Npy-hrGFP* mice received a co-injection of AAV-Fos-CreERT2 and AAV8-EF1a-DIO-hChR2(H134R)-mCherry viruses in the caudomedial NTS. Three weeks later, the mice were exposed to 3 experimental inductions, each separated by 96 h (Figure 3A). During each of these, the mice were fasted for 4 h during the light phase and received a bilateral NTS injection of aCSF or leucine followed 80 min later by an i.p. Injection of 40 mg/kg 4-OHT (hereafter designated as aCSF_induced_ and Leu_induced_ mice, respectively). Access to food was restored 4 h later to avoid food-induced Cre recombination. Mice were sacrificed 14 days after the last NTS injection. Using mCherry immunodetection in brain tissues, we characterized the neuroanatomical distribution of axonal projections and synaptic terminals of NTS leucine-sensing neurons. mCherry expression was dense in the caudomedial NTS of mice induced with NTS leucine injections, confirming the success of the approach (Figure. 3B). We did not detect mCherry^+^ signals in the ARH of Leu_induced_ mice, indicating that NTS leucine-sensing neurons do not project directly to the ARH (Figure. 3C). In contrast, we found mCherry^+^ fibers and terminals in the PVH and ventral DMH of the Leu_induced_ mice compared to controls (Figure 3D-E). Thus, NTS leucine-sensing neurons project to the PVH and the DMH.

The PVH and DMH are both good candidates to relay NTS leucine-sensing inputs from PrRP^NTS^ neurons to AgRP neurons. Both the PVH and DMH receive dense projections from TH^NTS^ neurons (Suppl. Fig. 3a–3c), are innervated by PrRP^+^ fibers, and express GPR10, the receptor for PrRP [42]. Previous monosynaptic retrograde tracing studies identified the PVH and DMH as the main sources of pre-synaptic inputs to AgRP neurons [43], and channelrhodopsin-assisted circuit mapping studies showed that all PACAP^PVH^ and LepR^DMH^ neurons project to and directly regulate the activity of AgRP neurons [43,44]. However, there is limited understanding of how these neuronal inputs to AgRP neurons may be engaged under physiological conditions to modulate appetite. To examine the role of PACAP^PVH^ and LepR^DMH^ in relaying leucine-sensing information from the NTS to AgRP neurons, we first used RNAscope to colocalize *Fos*, *Adcyap1* (transcript for PACAP), and *Prlhr* (transcript for PrRP receptor) or *Fos*, *LepR*, and *Gpr10* in the PVH and DMH respectively of mice that received NTS aCSF or leucine as previously described (Figure. 1A). These experiments confirmed that NTS leucine produces a significant increase in the number of *Fos-*expressing neurons in the PVH compared to vehicle injection (Suppl. Fig. 3d), but NTS leucine did not increase the number of *Prlhr*^*+*^, *Adcyap1*^+^, or *Prlhr*^*+*^/*Adcyap1*^+^ PVH neurons expressing *Fos* (Suppl. Fig. 3d–3e). In the DMH, NTS leucine increased *Fos* expression in a group of neurons concentrated in the caudal DMH (Figure. 3F). Overall, 30% of the DMH neurons co-expressed *LepR* and *Prlhr* (Suppl. Fig. 3g), and NTS leucine significantly increased the number of DMH *Prlhr*^+^, *Lepr*^+^, and *Lepr*^+^/*Prlhr*^+^ neurons expressing *Fos* (Figure 3F-H). Thus, NTS leucine activates neurons in the DMH that are well positioned to receive inputs from NTS PrRP neurons and project to AgRP neurons.

To confirm that the DMH relays NTS leucine-sensing inputs to AgRP neurons, we conducted activity-dependent circuit mapping from DMH neurons activated by NTS leucine. We delivered AAV-Fos-CreERT2 and AAV8-EF1a-DIO-hChR2(H134R)-mCherry viruses into the DMH of the *NPY-hrGFP* mice and exposed them to the similar induction paradigm previously used (Figure. 3I) to label axons and synaptic terminals of DMH neurons activated by NTS leucine. In the presence of 4-OHT, NTS leucine induced a significant increase in the number of neuronal cell bodies labeled with mCherry in the DMH (Figure 3J,K), confirming the success of the approach to label DMH neurons responsive to NTS leucine. We observed mCherry-labeled axons in the ARH (Figure. 3l) but failed to identify additional mCherry labeling in other brain regions (not shown). These data indicate that DMH neurons activated by NTS leucine provide axo-somatic innervation of the ARH. The projection field labeled with mCherry overlapped with the hrGFP immunofluorescent labeling of NPY/AgRP neurons (Figure. 3L). Analysis of mCherry^+^ puncta contacting ARH NPY-GFP neurons suggested that DMH leucine-sensing neurons were synaptically connected to AgRP neurons (Figure 3L-N). This claim was further supported by 3D immunofluorescent imaging analysis indicating that the contact surfaces between mCherry ^+^punta/fibers and NPY-GFP cells also colocalized with a presynaptic protein marker synaptophysin (SYP), suggesting the presence of synaptic contacts (Suppl. Fig. 3h–3i). Thus, NTS leucine activates DMH neurons that project to AgRP neurons.

#### 3.4 DMH neurons responsive to NTS leucine sensing are necessary for the anorectic effect of NTS leucine and high-protein diets and the long-term control of energy balance

**Figure 4:**
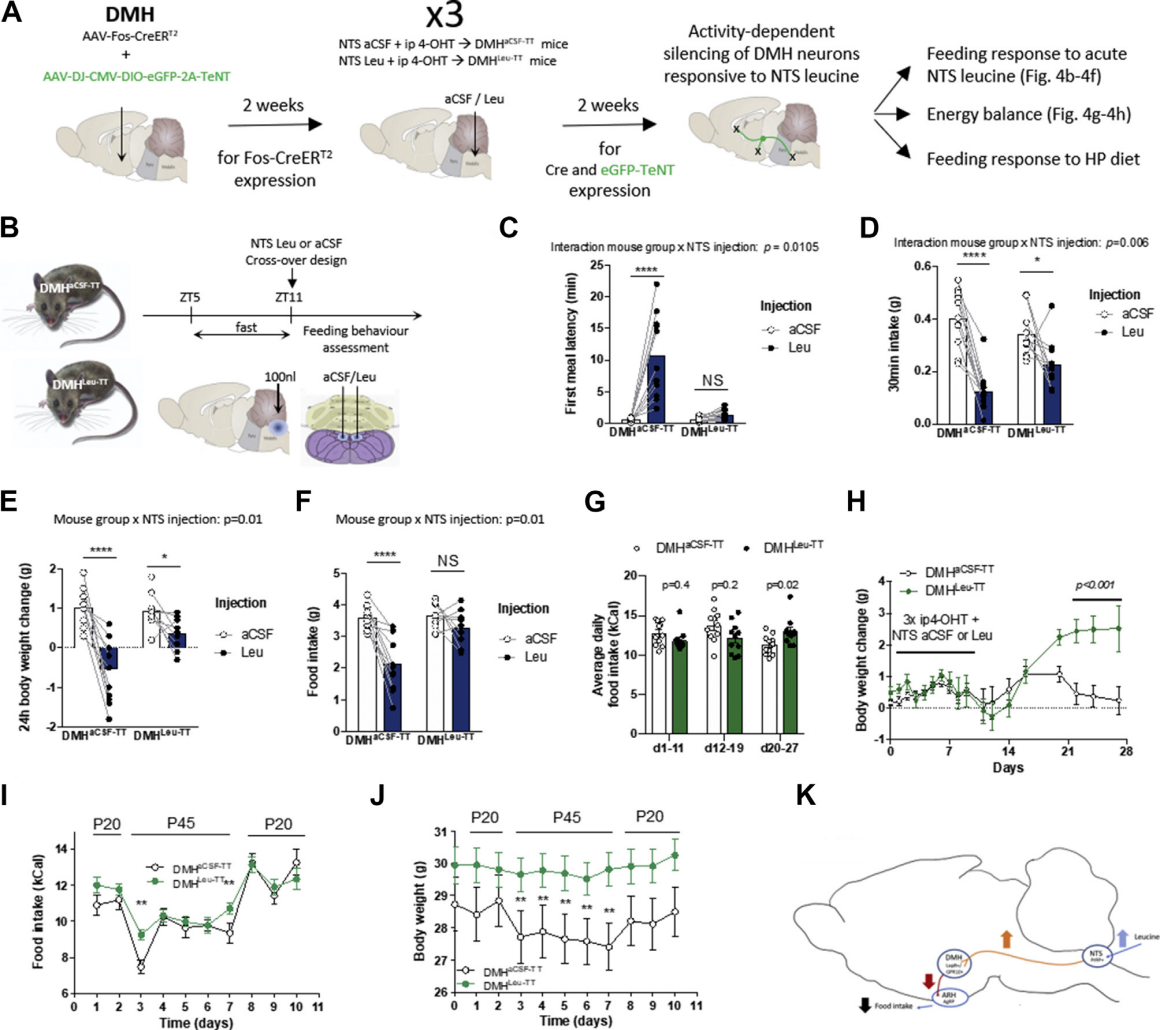
DMH neurons responsive to NTS leucine sensing are necessary for the anorectic effect of NTS leucine and high-protein diets. Diagram of the experimental paradigm to selectively silence DMH neurons receiving inputs from NTS leucine-sensing neurons (a, the DMH^aCSF−TT^ and DMH^Leu−TT^ mice). Diagram of the experimental paradigm used to test the feeding effect of NTS leucine in the DMH^aCSF−TT^ and DMH^Leu−TT^ mice (b). First meal latency (c), first meal size (d) 24 h body weight change (e), and 24 h food intake (f) in the DMH^aCSF−TT^ and DMH^Leu−TT^ mice following an acute injection of aCSF or leucine into the NTS. Average food intake (g) and weight change (h) of the DMH^aCSF−TT^ and DMH^Leu−TT^ mice during the 4 weeks following DMH injection with the Tet-Tox virus. Average food intake (i) and body weight (j) in the DMH^aCSF−TT^ and DMH^Leu−TT^ mice during transitions from diets containing 20% or 45% of energy as proteins. A schematic summary of the PrRP^NTS^ → LepR/GPR10^DMH^ → AgRP^ARH^ circuit mediating NTS leucine anorexic effects (k). All the results are shown as means ± SEM. ∗p < 0.05, ∗∗p < 0.01, ∗∗∗p < 0.001, and ∗∗∗p < 0.0001 vs aCSF or control group.

To directly test the role of DMH neurons engaged downstream from NTS leucine sensing in the appetite-suppressing effect of NTS leucine, we selectively silenced DMH neurons activated by NTS leucine using the cell-specific expression of tetanus toxin (TT) to prevent synaptic neurotransmitter release. To achieve this, we co-injected AAV-Fos-CreERT2 and AAV-DJ-CMV-DIO-eGFP-2A-TeNT into the DMH of the wild-type mice and exposed to the same induction paradigm as previously described via NTS injections of aCSF or leucine (DMH^aCSF−TT^ and DMH^Leu−TT^, respectively) (Figure 4A). We then compared the anorectic response to NTS leucine in the DMH^Leu−TT^ and DMH^aCSF−TT^ mice (Figure. 4B). In DMH^aCSF−TT^ controls, NTS leucine produced the expected behavioral response, including an increase in meal latency and a decrease in food intake following the injection (Figure 4C,D, Suppl. Movie 1, and Suppl. Fig. 4a and b). In contrast, NTS leucine failed to increase meal latency and decrease food intake in DMH^Leu−TT^ mice (Figure 4C,D, Suppl. Movie 2, and Suppl. Fig. 4a and 4b). Thus, DMH neurons engaged by NTS leucine sensing are required for the acute effects of NTS leucine on meal initiation and satiation. In addition, NTS leucine-induced reductions in 24 h food intake and 24 h weight change were blunted in the DMH^Leu−TT^ mice (Figure 4E,F), supporting a role for the NTS → DMH leucine-sensing circuit in the long-term feeding and metabolic consequences of NTS leucine sensing. Of note, over time, the DMH^Leu−TT^ mice developed a slight but significant hyperphagia (Figure. 4G) and gained significantly more weight than the DMH^aCSF−TT^ controls (Figure. 4H), supporting a role for DMH neurons receiving NTS leucine-sensing inputs in the chronic maintenance of energy balance.

Supplementary video related to this article can be found at https://doi.org/10.1016/j.molmet.2020.101070

The following is/are the supplementary data related to this article:

Suppl. Movie 1 Video recording of a DMH^Leu−TT^ mouse following NTS aCSF administration and food presentation (paradigm shown in Fig. 1a). Suppl. Movie 1

Suppl. Movie 2 Video recording of a DMH^Leu−TT^ mouse following NTS Leu administration and food presentation (paradigm shown in Fig. 1a). Suppl. Movie 2

Next, we assessed whether this newly characterized circuit was relevant not only for the feeding-suppressive effect of NTS leucine, but also for the anorectic response to high-protein feeding. In mice, acute exposure to a high-protein diet reduces appetite and weight gain [45]. While the central mechanisms mediating these responses are poorly characterized, the NTS is established as a neuroanatomical site responding to high-protein diets [46]. Furthermore, a single high-protein meal is sufficient to increase brain leucine concentration [46], supporting the possibility that NTS leucine-sensing neurons could mediate appetite suppression in response to dietary proteins. To address this, we exposed the DMH^Leu−TT^ and DMH^aCSF−TT^ mice to a high-protein diet containing 45% of energy as protein (P45) and isocaloric with a control maintenance diet containing 20% energy as protein (P20, control maintenance diet). To avoid a neophobic response to the P45 diet, the mice were first briefly exposed to P45 pellets (3 times for 30 min on 3 consecutive days). A week later, the mice were switched to the P45 diet for 6 days. In the control DMH^aCSF−TT^ mice, the P45 diet produced a rapid 30% decrease in energy intake, followed by a sustained 10–15% reduction in daily energy intake in the following days (Figure 4I and Suppl. Fig. 4c). The anorectic response to the high-protein diet was associated with sustained weight loss (Figure 4J and Suppl. Fig. 4d), confirming the feeding and metabolic effects of the P45 diet under these conditions. In contrast, the anorectic response to P45 was blunted in the DMH^Leu−TT^ mice (Figure 4I and Suppl. Fig. 4c) and remarkably, the P45 diet did not produce a weight response in these mice (Figure 4J and Suppl. Fig. 4d). Thus, DMH neurons activated by NTS leucine are required for the acute anorectic response to high-protein diets, while other pathways likely mediate the sustained anorectic effect of dietary proteins. Unexpectedly, these results indicate that the NTS → DMH leucine-sensing circuit contributes to the metabolic effect of high-protein diets. These results provide a central mechanism for the behavioral and metabolic responses to dietary proteins.

We then examined the neuronal activation patterns of various AgRP output areas in the DMH^aCSF−TT^ and DMH^Leu−TT^ mice after an acute terminal NTS leucine injection with the paradigm outlined in Figure 1A. We observed approximately twice as many eGFP-TeNT-expressing cells in the DMH^Leu−TT^ over DMH^aCSF−TT^ mice while the NTS leucine-induced DMH activation patterns were comparable between the two groups, indicating the success of our targeting strategy, which did not appear to affect the responsiveness of DMH neurons to NTS leucine sensing (Suppl. Fig. 4e–4f). Of note, approximately one-half of all of the eGFP-TeNT-labeled DMH cells were activated by the terminal NTS leucine injection in the DMH^Leu−TT^ mice while only approximately less than 10% of the labeled cells were activated by the aCSF injection (Suppl. Fig. 4g), validating the strategy's specificity. As expected, the inhibitory effects of the NTS leucine injection on AgRP^ARH^ neurons was blunted in the DMH^Leu−TT^ mice (Suppl. Fig. 4h). We then analyzed neuronal activation in a range of previously identified AgRP output areas [47]. Consistent with ARH activation patterns, the inhibitory effects of NTS leucine on the paraventricular nucleus of the thalamus (PVT) and lateral hypothalamus (LH) were blunted in the DMH^Leu−TT^ mice (Suppl. Fig. 4h). However, we did not observe significant modifications of NTS leucine-induced PVH activation, demonstrating the complexity of the relative contributions to those brain areas receiving influences from both the NTS and AgRP^ARH^ (Suppl. Fig. 4h). We did not observe robust c-Fos expression in the anterior bed nucleus of the stria terminalis (aBNST) and central amygdala (CeA) across all of our conditions in our mild 6 h fasting paradigm (Suppl. Fig. 4i). Together, these results revealed the efficacy of NTS leucine sensing in regulating the downstream targets of AgRP^ARH^ neurons and corroborating the role of DMH as an inhibitory relay between NTS leucine sensing neurons and AgRP neurons.

## 4 Discussion

Our findings reveal a mechanism through which nutrient sensing in the NTS regulates food-seeking behavior, satiety, and long-term energy balance through polysynaptic inhibition of AgRP neurons. We demonstrate that PrRP^NTS^ neurons engage this circuit in response to the detection of the branched-chain amino acid leucine, a signal of dietary protein availability. Silencing of DMH neurons responsive to NTS leucine sensing blunts leucine's appetite-suppressive effects and dampens the anorexic and weight loss responses to a high-protein diet, extending the role of this circuit in the behavioral and metabolic responses to dietary proteins.

Our data expand the characterization of the functional diversity of NTS TH neurons to include a subset of neurons expressing PrRP and CTR, projecting to the DMH and modulating feeding initiation and satiety via downstream projections to AgRP neurons. While other neuronal populations (including NTS and PVH neurons) activated by NTS leucine are likely involved in other behavioral, metabolic, and neuroendocrine outputs of NTS leucine sensing, the circuit described in this report is sufficient to entirely explain the appetitive consequences of NTS leucine detection. These results support a model in which specialized neuronal populations regulate specific behavioral outputs. Intriguingly, although PrRP and CTR neurons of the NTS have been shown to project to the PBN [42,48], this site is not activated by NTS leucine, suggesting further functional diversity among these neurons. This highlights the relevance of the activity-dependent labeling and circuit-mapping strategy for the characterization of functionally relevant brain relays connecting NTS leucine-sensing neurons and AgRP neurons.

Recent research indicated that AgRP neurons integrate various sensory inputs including environmental food-related cues and visceral mechanosensory and nutritional inputs [18,[49], [50], [51]]. Our research extends the integrative capability of AgRP neurons to include brainstem nutrient-sensing inputs. Given that visceral vagal afferents terminate in the caudomedial NTS where PrRP/CTR neurons are concentrated, it is likely that intestinal sensory inputs engage the same circuit as NTS leucine to inhibit AgRP neurons. Thus, the PrRP^NTS^ → LepR^DMH^ circuit may be specialized in integrating nutritional cues arising from multiple central and peripheral interoceptors. These sites may be only partially functionally redundant given the indication that caloric density is the primary information carried by vagal afferents activated by nutrients in the gut [17], whereas NTS nutrient sensing surveys nutritional status and postabsorptive nutrient availability. With the ability to monitor the availability of specific nutrients and relay this information to forebrain centers mediating the long-term control of energy balance, PrRP ^NTS^ neurons and their projections to LepR^DMH^ neurons are well positioned to contribute to the production of nutrient-specific satiety, a well-established behavior lacking mechanistic characterization [52].

DMH^LepR^ neurons also process environmental food-related cues in the regulation of AgRP activity, which may be integrated with former signals as well [44]. To the best of our knowledge, these are the only inhibitory inputs to AgRP neurons identified to date. However, polysynaptic retrograde tracing from AgRP neurons in this study revealed additional medullary inputs to AgRP neurons that may extend the inhibitory control of this population.

While the novel circuit we identified in this study provides new insights into hindbrain ascending pathways for the control of feeding, additional research is needed to clarify how the NTS integrates distinct food-related signals to produce a variety of behavioral responses. In particular, whether NTS leucine-responsive cells are also responsive to other nutrients such as glucose and free fatty acid remains to be determined.

Collectively, this study resolves the mechanisms through which NTS nutrient-sensing modulates food-seeking behavior and provides insights into the functional organization feeding regulatory circuits, creating new opportunities for the treatment of hyperphagic obesity and related metabolic disorders.

## Author contributions

AHT conducted the experiments, analyzed the data, and drafted the manuscript. DN conducted the experiments and data analysis. TD and HS conducted the experiments. CB designed and conducted the experiments, analyzed the data, and prepared the manuscript.

## Materials and correspondence

Correspondence and material requests should be addressed to Clemence Blouet.

## Coflict of interest

None declared.
