# Supplementary material for: Nutrient sensing in the nucleus of the solitary tract mediates non-aversive suppression of feeding via inhibition of AgRP neurons
Source: Mol Metab. 2020 Sep 6;42:101070. doi: 10.1016/j.molmet.2020.101070 (PMC7549147; doi:10.1016/j.molmet.2020.101070)
Supplement: Multimedia component 1 [file mmc1.pdf]

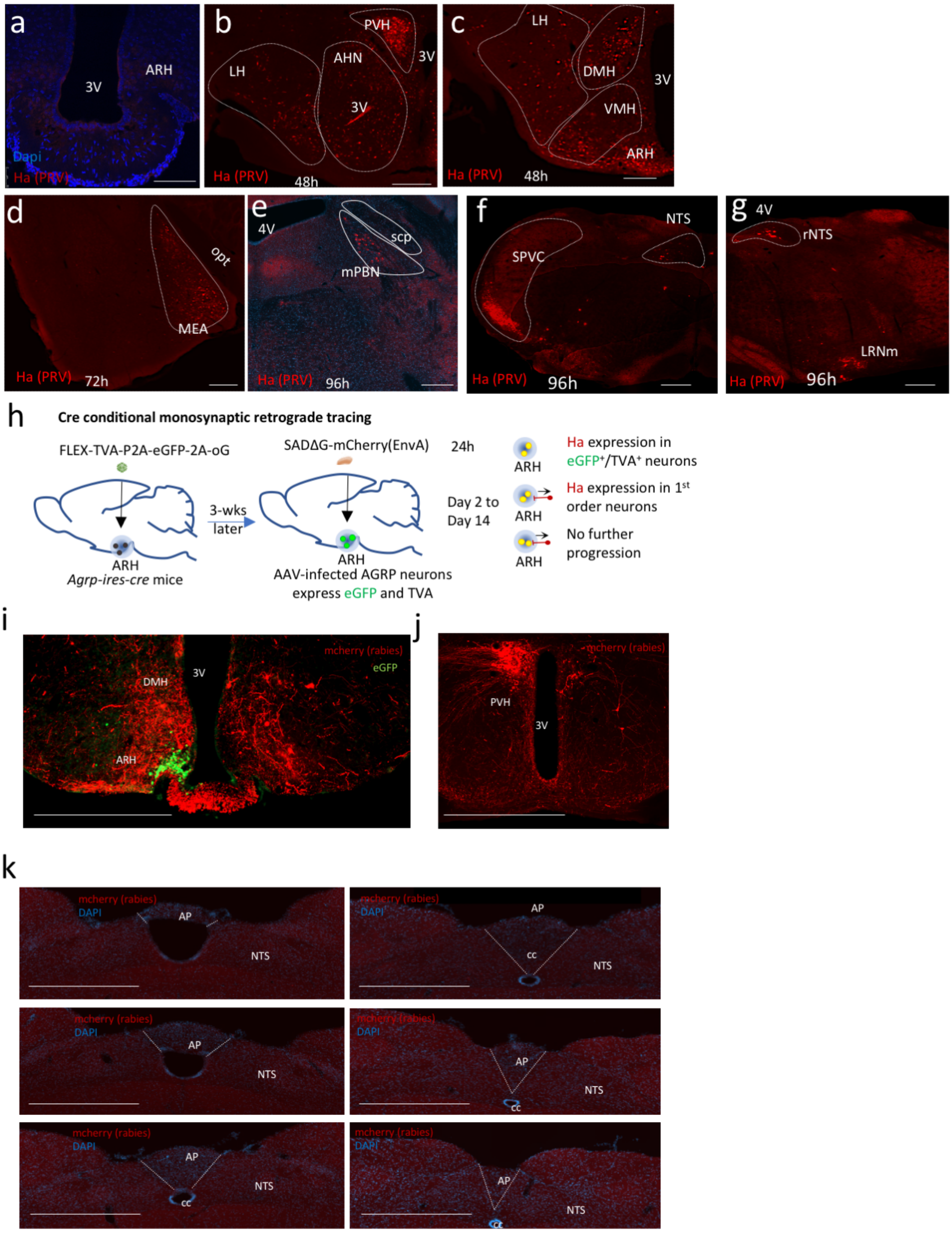

Suppl. Fig. 1: Cre-dependent retrograde polysynaptic and monosynaptic viral tracing in *Agrp-ires-cre* mice. HA immunodetection in WT mice 96h post inoculation (a) and in *Agrp-ires-cre* mice 48h (b, c) 72h (d) and 96h (e, f, g) after a bilateral injection of PRV-Introvert in the ARH (Scale bar: 400um). Protocol for Cre conditional monosynaptic retrograde tracing using SADΔG-mCherry(EnvA) (h) and expression of the mCherry and eGFP in the hypothalamus (i, j) and hindbrain (k) of *Agrp-ires-cre* mice unilaterally infected with rAAV8-hSyn-FLEX-TVA-P2A-eGFP-2A-oG and SADΔG-mCherry(EnvA) 2-weeks after rabies infection (Scale bar: 800um). 3V: 3rd ventricle, ARH: arcuate nucleus of the hypothalamus, PVH: paraventricular hypothalamic nucleus, DMH: dorsomedial hypothalamic nucleus, LH: lateral nucleus of the hypothalamus, VMH: ventromedial hypothalamic nucleus, opt: optical tract, MEA: medial amygdala, mPBN: medial parabrachial nucleus, scp: superior cerebellar peduncle, AHN: anterior hypothalamic nucleus, NTS: nucleus of the solitary tract, AP: area

postrema, cc: central canal, rNTS: rostral nucleus of the solitary tract, SPVC: Spinal nucleus of the trigeminal, LRNm: Lateral reticular nucleus, magnocellular part.

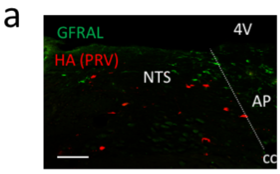

Supp. Fig. 2: GFRAL and HA immunolabelling in the NTS of *Agrp-ires-cre* mice 96h after PRV-Introvert delivery into the ARH. Scale bar: 200um. 4V: 4th ventricle. AP: area postrema, NTS: nucleus of the solitary tract.

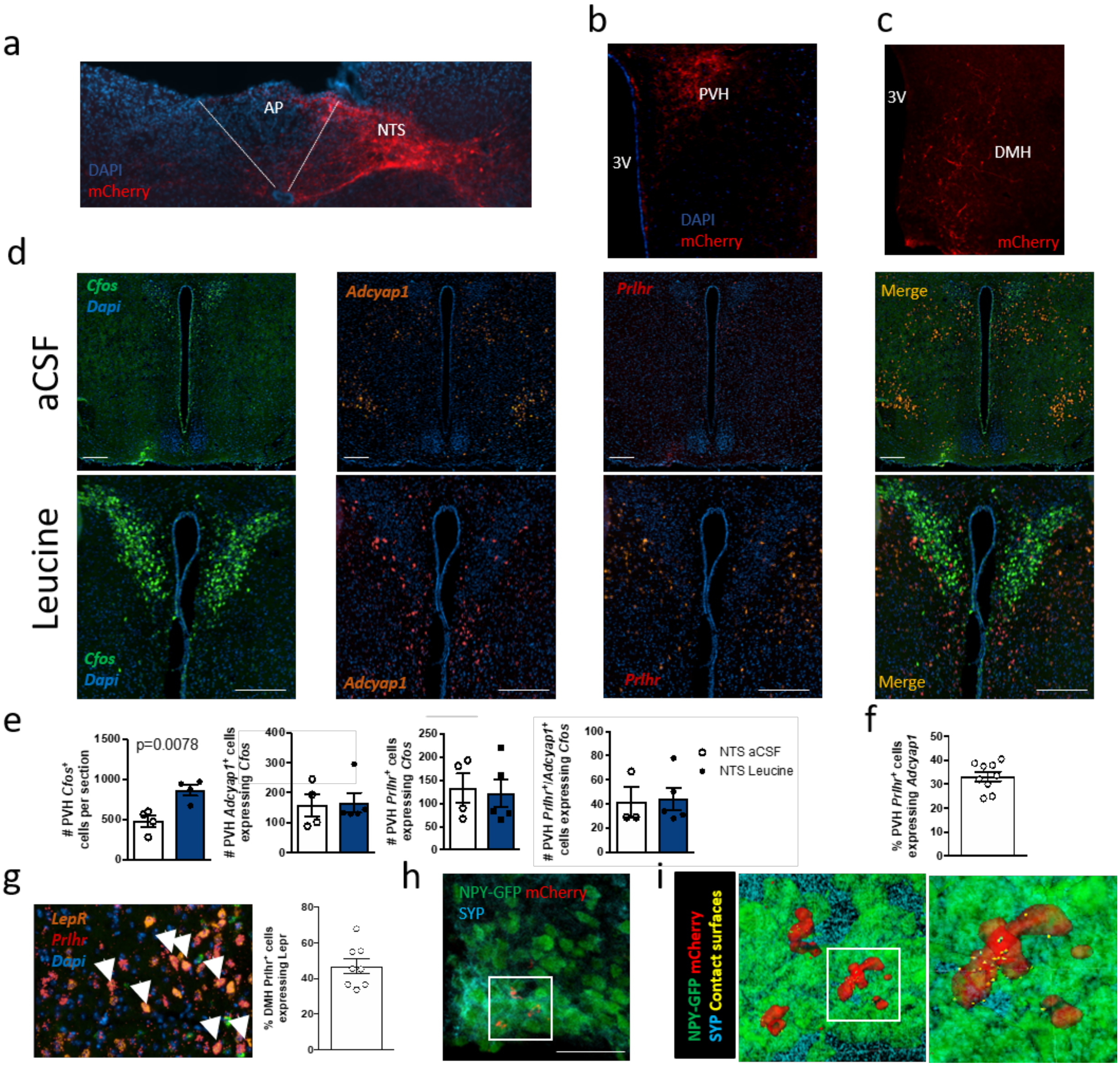

Supp. Fig. 3: Representative images on mcherry expression in the NTS (a), PVH (b) and DMH (c) in *Th-cre* mice that received a unilateral injection of AAV8-EF1a-DIO-hChR2(H134R)-mCherry into the NTS to label synaptic terminals of NTS TH neurons. Representative images (d) and quantification (e) of *Cfos*, *Adcyap1* and *Prlhr* expression in the PVH of mice

exposed to NTS aCSF or Leucine. Quantification of *Prlhr* and *Adcyap1* co-expression in the PVH (f). Representative image and quantification (g) of Prlhr and *LepR* co-expression in the DMH. Scale bar is 200um. Representative image (h) and Imaris 3D reconstruction (i) of mCherry and synaptophysin (SYP) immunolabelling in the ARH of *Npy-hr-GFP* mice mice following injection of AAV-Fos-CreERT2 and AAV8-EF1a-DIO-hChR2(H134R)-mCherry viruses in the DMH and inductions with NTS aCSF or leucine. Scale bar is 200um. All results are shown as means ± SEM.

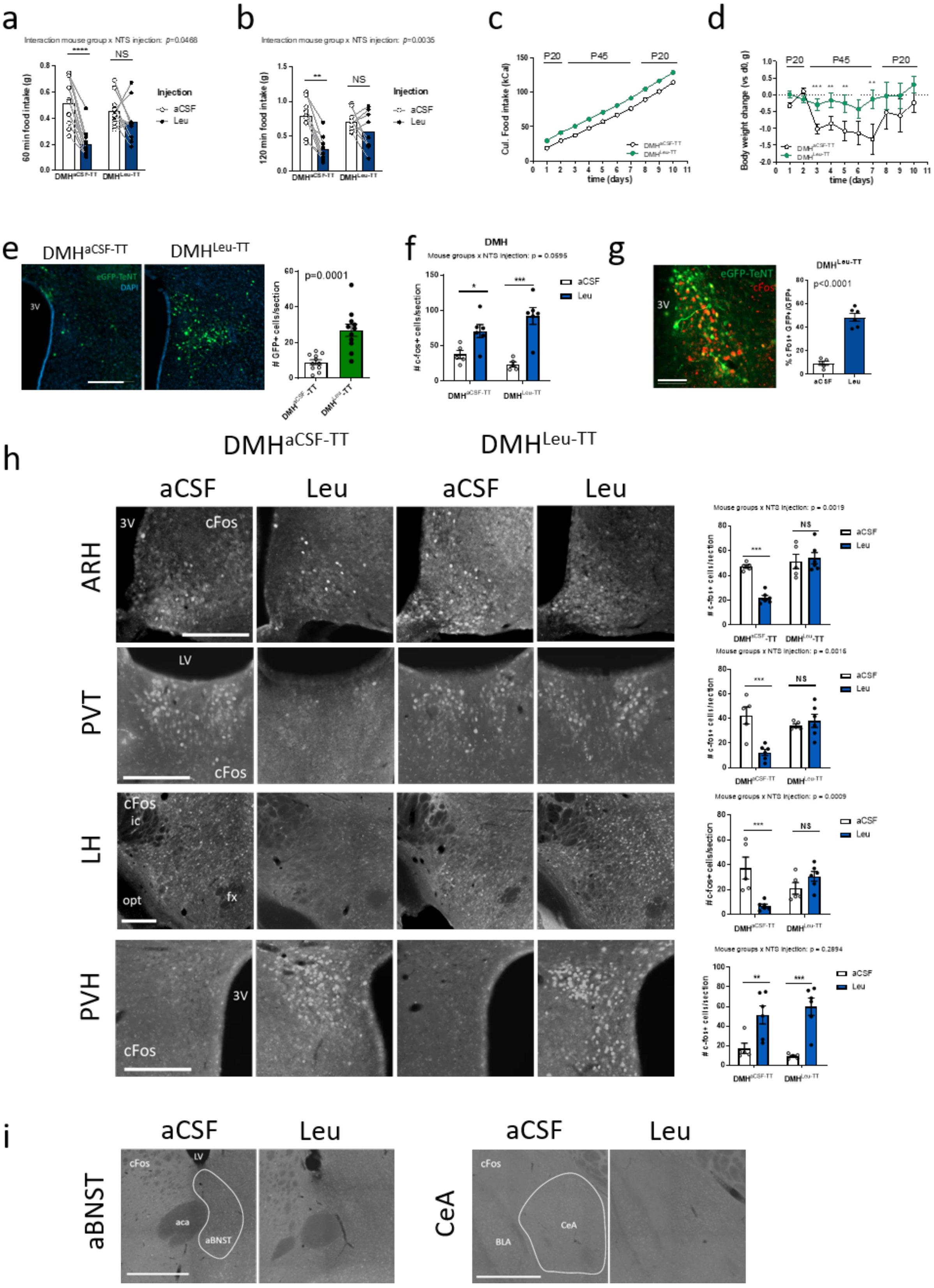

Supp. Fig. 4: 60min (a) and 120 min (b) food intake in DMH$^{aCSF\text{-}TT}$ and DMH$^{leu\text{-}TT}$ mice following an acute injection of aCSF or leucine into the NTS. Culminative food intake (c) and body weight change (d) in DMH$^{aCSF\text{-}TT}$ and DMH$^{leu\text{-}TT}$ mice during transitions from diets containing 20% or 45 % of energy as proteins. Representative images and quantification of eGFP-TeNT positive cells in the DMH of DMH$^{aCSF\text{-}TT}$ and DMH$^{leu\text{-}TT}$ mice after activity-dependent inductions (e). Quantification of c-fos positive cells in the DMH of DMH$^{aCSF\text{-}TT}$ and DMH$^{leu\text{-}TT}$ mice after acute NTS leucine injection (f). Representative image

and quantification of relative c-fos expression in eGFP-TeNT positive cells in the DMH of DMH$^{leu\text{-}TT}$ mice after NTS aCSF or leucine injection (g). Representative images and quantification of c-fos positive cells in the ARH, PVT, LH and PVH of DMH$^{aCSF\text{-}TT}$ and DMH$^{leu\text{-}TT}$ mice after terminal acute NTS leucine injection (h). Representative images of the lack of robust c-fos expression in aBNST and CeA across conditions in the NTS injection paradigm (i).
