# Supplementary material for: Nutrient sensing in the nucleus of the solitary tract mediates non-aversive suppression of feeding via inhibition of AgRP neurons
Source: Mol Metab. 2020 Sep 6;42:101070. doi: 10.1016/j.molmet.2020.101070 (PMC7549147; doi:10.1016/j.molmet.2020.101070)
Supplement: Multimedia component 2 [file mmc2.docx]

**Supp. Movie 1**: Video recording of a DMH^leu-TT^ mouse following NTS aCSF administration and food presentation (paradigm shown on Fig.1a).

**Supp. Movie 2**: Video recording of a DMH^leu-TT^ mouse following NTS Leu administration and food presentation (paradigm shown on Fig.1a).

**Supplemental Table 1**: Modified diets composition

|  | **P20** |  | **P45** |  |
| --- | --- | --- | --- | --- |
| Ingredient | gm |  | gm |  |
| Casein | 233 |  | 510 |  |
| L-Cystine | 3.49 |  | 7.65 |  |
| Corn Starch | 326.6 |  | 156.5 |  |
| Maltodextrin 10 | 150 |  | 75 |  |
| Sucrose | 107.1 |  | 107.1 |  |
| Cellulose | 50 |  | 50 |  |
| Soybean Oil | 88.9 |  | 88.9 |  |
| tBHQ | 0.014 |  | 0.014 |  |
| Mineral Mix S10022G | 0 |  | 0 |  |
| Mineral Mix S10022C | 3.5 |  | 3.5 |  |
| Calcium Carbonate | 10 |  | 12.35 |  |
| Calcium Phosphate, Dibasic | 3.4 |  | 0 |  |
| Potassium Citrate, 1 H20 | 3 |  | 8 |  |
| Potassium Phosphate, Monobasic | 6.31 |  | 0 |  |
| Sodium Chloride | 2.59 |  | 2.59 |  |
| Vitamin Mix V10037 | 10 |  | 10 |  |
| Choline Bitrartrate | 2.5 |  | 2.5 |  |
| FD&C Yellow Dye #5 | 0 |  | 0 |  |
| FD&C Red Dye #40 | 0.05 |  | 0 |  |
| FD&C Blue Dye #1 | 0 |  | 0.05 |  |
| Total | 1000.4 |  | 1034.1 |  |
|  |  |  |  |  |
|  | gm | kcal | gm | kcal |
| Protein | 206 | 824.8 | 451 | 1805.4 |
| Carbohydrate | 594 | 2375 | 349 | 1394 |
| Fat | 89 | 800.2 | 89 | 800.2 |
| Fiber | 50 | 0 | 50 | 0 |
| Total | 939 | 4000 | 939 | 4000 |
|  |  |  |  |  |
|  | gm% | kcal% | gm% | kcal% |
| Protein | 21 | 21 | 44 | 45 |
| Carbohydrate | 59 | 59 | 34 | 35 |
| Fat | 9 | 20 | 9 | 20 |
